# On the History and Applications of Congenic Strains in *Cryptococcus* Research

**DOI:** 10.3390/pathogens9090750

**Published:** 2020-09-15

**Authors:** Benjamin J. Chadwick, Xiaorong Lin

**Affiliations:** 1 Department of Plant Biology, Franklin College of Arts and Sciences, University of Georgia, Athens, GA 30602, USA; Benjamin.Chadwick@uga.edu; 2 Department of Microbiology, Franklin College of Arts and Sciences, University of Georgia, Athens, GA 30602, USA

**Keywords:** *Cryptococcus*, congenic pair, mating type locus, morphogenesis, neurotropism, QTL mapping, genetic linkage, mitochondrial inheritance

## Abstract

Congenic strains have been utilized in numerous model organisms to determine the genetic underpinning of various phenotypic traits. Congenic strains are usually derived after 10 backcrosses to a recipient parent, at which point they are 99.95% genetically identical to the parental strain. In recent decades, congenic pairs have provided an invaluable tool for genetics and molecular biology research in the *Cryptococcus neoformans* species complex. Here, we summarize the history of *Cryptococcus* congenic pairs and their application in *Cryptococcus* research on topics including the impact of the mating type locus on unisexual reproduction, virulence, tissue tropism, uniparental mitochondrial inheritance, and the genetic underpinning of other various traits. We also discuss the limitations of these approaches and other biological questions, which could be explored by employing congenic pairs.

## 1. Introduction

A major goal of genetics research is to determine the genes underpinning a phenotypic trait of interest. This is traditionally achieved through linkage mapping based on segregation among progeny derived from a cross between two individuals differing in the phenotype. This approach becomes challenging when individuals are genetically diverse or the phenotype of interest is controlled by the interaction of many genetic loci [1,2]. Genome-wide association studies (GWAS) analyze large populations to make correlative but not causative relationships between natural variants and phenotypes. To better understand the outcome of a variant, reducing diversity in other genetic loci is desired, which is what makes twin studies so powerful [3]. In model organisms, the tool that minimizes the genetic background noise is the construction of congenic pair strains. Congenic pair strains are typically generated from backcrossing to a parental strain for 10 generations, which renders ~99.95% genetic identity with that parent. Constructing a congenic pair allows the allele of interest to be introduced into a well-defined genome and reduce potential unknown background interference. A phenotype of interest can be selected during the backcrosses to maintain the causative allele(s). Therefore, congenic pair strains can be applied to identify variants responsible for an interesting trait or to examine genetic interactions. Manipulation of the genetic background of an organism using this method has been applied in mouse genetics since it was first published by George Snell in 1948 [4]. Snell used the backcrossing strategy to generate congenic mice differing in their response to tumor transplantation in order to discover histocompatibility genes. Congenic strains have been used in a wide range of model organisms for many purposes, including studying blood groups in the bird *Columbidae* [5], sucrose fermentation in the yeast *Saccharomyces* [6], protein variants in the protist *Tetrahymena* [7], or fitness in the fruit fly *Drosophila* [8].

In fungi, a congenic pair allows for the transfer of a mutation from one mating type to another while maintaining the same genetic background. Different single mutants generated in compatible mating types can be crossed to create double mutants in either mating type. A genetic linkage analysis can be performed by crossing a mutant strain with a congenic wild type partner to determine the link between the observed phenotype and the designated mutation. This experimental tool can be used to establish the causative relationship between a phenotype and a targeted mutation, or to identify mutants with interesting phenotypes linked to an unknown mutation isolated from genetic screens. Such tools boost the use of *Saccharomyces cerevisiae*, *Schizosaccharomyces pombe*, and *Aspergillus nidulans* as model organisms, and contribute to the rapid rise of *Cryptococcus neoformans* as a model for fungal pathogenesis studies.

## 2. Diversity of *Cryptococcus* Pathogenic Species

*Cryptococcus* is an environmental fungus. This fungus enters human lungs through inhalation and can be cleared or becomes dormant in immunocompetent individuals. However, this fungus can reactivate and disseminate through the bloodstream when the host is immunocompromised, often due to AIDS or immune-suppressive treatments for cancer or organ transplant. Due to its predilection to the brain, this fungus causes fatal cryptococcal meningitis. Cryptococcosis is responsible for over 15% of all AIDS-related deaths [9]. The *Cryptococcus* pathogenic species is a species complex, composed of distinct groups with great genetic and phenotypic diversity. The major pathogenic *Cryptococcus* species were originally separated into different serotypes (A, B, C, and D, or their hybrids) based on their capsular epitopes or biochemical properties tested by diagnostic media [10,11]. Serotypes A and D correspond to *C. neoformans* var. *grubii* and *C. neoformans* var. *neoformans*, while serotypes B and C refer to *C. gattii*. Different isolates of *C. neoformans* and *C. gattii* are further divided into molecular groups, based on their DNA sequencing information. In general, serotype A isolates have been separated into three molecular types referred to as VNI, VNII, and VNB (only Botswana isolates), and serotype D has been typed as VNIV. Isolates with the molecular type VNIII are serotype AD hybrids. Molecular types VGI, VGII, VGIII, VGIV, and VGV correspond to *C. gattii* isolates (see review [12]).

Significant genetic and phenotypic diversity are reported among the groups (many referenced here [13]). This is not unexpected given that serotype A and serotype D of *C. neoformans* diverged from a common ancestor an estimated 18.5 million years ago, and serotypes B and C of *C. gattii* diverged about 9.5 million years ago based on multi-locus sequence typing [14]. *C. gattii* and *C. neoformans* were estimated to have diverged roughly 37 million years ago. In comparison, humans and chimpanzees diverged about 12.1 million years ago, and humans and gorillas diverged from each other about 15.1 million years ago based on whole genome sequencing data [15]. Functional divergence of genetic networks between serotypes A and D of *C. neoformans* is not uncommon, as demonstrated in the Protein kinase A (PKA) and High-osmolarity glycerol (HOG) pathways, the two major stress-sensing pathways [16,17]. Even within the same serotype, great genetic heterogeneity exists. For instance, VNI and VNII of serotype A were estimated to have diverged from each other about 5 million years ago, and different *C. gattii* VG molecular types about 10 million years from each other [18].

## 3. The First *Cryptococcus* Congenic Pair

*C. neoformans* and *C. gattii* are ubiquitous basidiomycetes with a bipolar mating system, defined as either mating type (*MAT*) **a** or α [19]. Unlike *S. cerevisiae*, *Cryptococcus* does not have the ability to switch mating types [20]. Laboratory crosses between **a** and α isolates lead to a 1:1 segregation of the mating types in the progeny. However, in 1978, Kwon-Chung and Bennett found that the α mating type was 30–40 times more prevalent than the **a** mating type among the tested 105 environmental and 233 clinical isolates of *C. neoformans* [10]. Similar findings were later reported based on samples isolated from different locations [21,22,23,24]. This raised the question about the impact of the mating type locus on *Cryptococcus* biology and pathogenicity.

**Figure 1:**
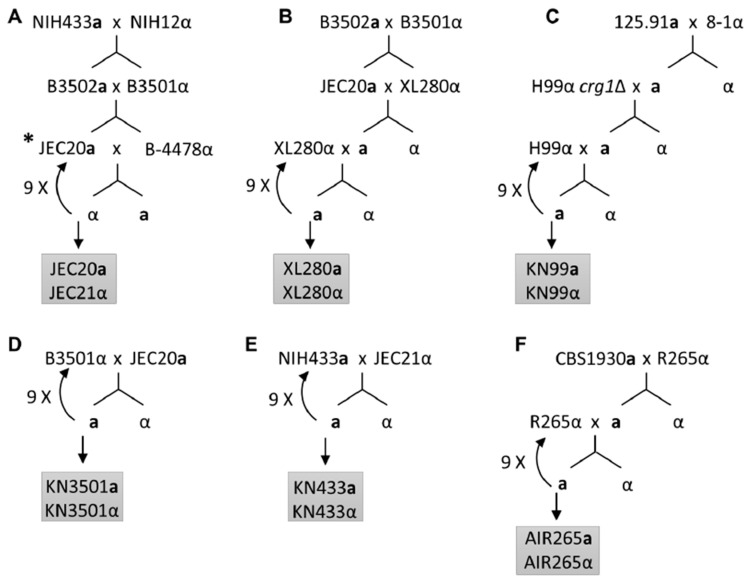
Schemes of current Cryptococcus congenic pairs. (**A**) NIH433**a** and NIH12α were crossed to generate B3502**a** and B3501α. B3502**a** and B3501α were crossed to generate JEC20**a** and B-4478α. These strains were crossed, and an α progeny was selected and backcrossed to JEC20**a**. Backcrossing was repeated for a total of 10 times to create the congenic pair JEC20**a**/21α. * B3502**a** may be identical to JEC20**a** based on two previous studies [34,35]. (**B**) JEC20**a** and XL280α, progeny of B3502**a** and B3501α, were crossed, and an **a** progeny was selected and backcrossed to XL280α. Backcrossing was repeated for a total of 10 times to create congenic pair XL280**a**/XL280α. (**C**) 125.91**a** and 8-1α were crossed, and an **a** progeny was selected to mate with H99α *crg1*Δ. An **a** progeny without the *CRG1* mutation was then crossed with H99α. Backcrosses were continued for a total of 10 times to create congenic strains KN99**a** and KN99α. (**D**) B3501α was crossed with JEC20**a**, and an **a** progeny was selected to backcross to B3501α. Backcrosses were repeated for a total of 10 times to generate congenic strains KN3501**a** and KN3501α. (**E**) NIH433**a** and JEC21α were crossed, and an **a** progeny was selected to backcross to NIH433**a**. Backcrosses were repeated for a total of 10 times to generate congenic strains KN433**a** and KN433α. (**F**) CBS1930**a** was crossed with R265α. An **a** progeny was selected to backcross to R265α, and backcrosses were repeated for a total of 10 times to yield AIR265**a** and AIR265α.

Congenic strains differing primarily at the mating type locus in multiple genetic backgrounds would be valuable tools to address such questions. Kwon-Chung and her colleagues developed the first congenic pair [25]. They used progeny of serotype D strains NIH12 and NIH433, a clinical α and an environmental **a** isolate, as the starting strains. These strains, named B-3501 and B-3502, had slightly different karyotypes based on contour-clamped homogenous electric-field (CHEF) electrophoresis. Their cross generated B-4476(**a**), which was used as the parent strain in this congenic pair construction. After six backcrosses to B-4476, the karyotype differences were no longer observed between progeny and parent. A total of 10 backcrosses in total were completed to create the first congenic pair now known as JEC20**a** and JEC21α, with JEC20**a** as the new alias for B-4476 (Figure 1A). The authors found that α progeny from a cross between the congenic pair strains JEC20 and JEC21 were more virulent than **a** progeny in an intravenous infection model of murine cryptococcosis. Because these strains primarily differ in the mating type locus, this suggests that the mating type locus is linked to virulence.

The establishment of this congenic pair also led to the cloning of the mating type locus and the pheromone genes, as well as the discovery of the MAPK pheromone sensing and response pathway controlled by the genes encoded by the *MAT* locus [26]. Moore and Edman also showed there was only a single mating type locus in *C. neoformans* [26]. This contrasts with the existence of alternate mating type loci in the same genome as seen in *S. cerevisiae*, which allows the budding yeast to switch mating types [20]. JEC21α later became the first *Cryptococcus* strain to be whole genome sequenced and extensively used in genetic manipulations to study gene function at the molecular level in this organism [27].

## 4. Summary of the Current Congenic Pairs in *Cryptococcus*

The *C. neoformans* species complex is responsible for about 99% of total cryptococcosis cases globally [12], and so it is no surprise that five of the six congenic pairs have been constructed in the *C. neoformans* background (Table 1). After the Vancouver Island outbreak caused by *C. gattii* starting from 1999, hundreds of *C. gattii* isolates have been discovered in Canada and the VGII type is now the most frequently reported in Northwest America [28]. The heightened virulence of the Vancouver outbreak strains motivated the construction of the first VGII background congenic pair based on the sequenced strain R265 [29]. The six current *Cryptococcus* congenic pairs are summarized in Table 1: JEC20**a**/JEC21α (VNIV), KN99**a**/α (VNI), KN433**a**/α (VNIV), KN3501**a**/α (VNIV), XL280**a**/α (VNIV) and AIR265**a**/α (VGII).

These congenic pairs differ widely in various phenotypes, including capsule production, melanization, filamentation, mating, or their virulence in the murine models of cryptococcosis (Table 1). Congenic pairs KN99**a**/α and AIR265**a**/α are among the most aggressive strains as they kill mice around 3–4 weeks following intranasal infections or about one week following intravenous infection. Inoculum only has modest effects on the median days of survival of infected animals. XL280**a**/α are modestly lower in virulence than KN99**a**/α and AIR265**a**/α strains, but this pair are the most virulent among the serotype D congenic pairs including JEC20**a**/21α, KN3501**a**/α, and KN433**a**/α. The intranasal mouse infection model using JEC20**a**/21α requires high inoculum (e.g., 1 × 10^7^) and months for mortality studies, and even then some mice can survive the infection [30,31]. Thus, the intravenous infection model that bypasses the initial lung infection is often preferred when JEC20**a**/21α or other serotype D congenic pairs such as KN3501**a**/α and KN433**a**/α are used. XL280**a**/α are far more virulent than these related serotype D pairs, and XL280**a**/α are used in both the intranasal and intravenous infection models [32]. Although all the serotype D congenic pairs are excellent maters, XL280**a**/α exhibit the most robust self-filamentation in vitro (monokaryotic fruiting or unisexual reproduction), which makes this pair an excellent model for morphogenesis investigation [33]. In contrast, JEC20**a**/21α are the better choice for investigation of bisexual mating due to minimal interference from unisexual reproduction.

There are still seven molecular types with no corresponding congenic pair. Construction of congenic pairs in these molecular types would provide a platform for functional comparative studies of this species complex to advance our understanding of their biology and pathogenicity.

## 5. Examine the Impact of Mating Type on Virulence and Tissue Tropism

Because of the dominance of the α mating type in 99% of cryptococcal natural isolates found in the environment and clinic, a major question of the field is whether the mating type α enhances fitness and virulence. Kwon-Chung and her colleagues took advantage of the first congenic pair to probe this question. They found that α and **a** progeny from a cross between JEC20**a** and JEC21α were comparable in growth in vitro, but α progeny were more virulent than their **a** siblings in the intravenous infection murine model [25]. Nielsen found that KN433α was modestly more virulent than its congenic partner KN433**a**, while KN3501α was comparable in virulence to its congenic partner KN3501**a** [34]. These congenic pairs are all related serotype D strains (Figure 1), with the α mating type locus originating from NIH12 and the **a** mating type locus originating from NIH433. The difference in virulence between **a** and α in these congenic strains is therefore likely due to the difference in the interaction between the mating type locus and their different genetic backgrounds.

The construction of serotype A congenic strains was not initiated until a decade later due to the lack of serotype A **a** isolates. Although it had been long suspected that serotype A **a** isolates exist given the presence of **a**ADα hybrid isolates, the first serotype A **a** strain, 125.91, was not discovered until 2000 [36]. Fortunately, this **a** strain, isolated from an AIDS patient in Tanzania, was able to mate with another serotype A clinical α strain 8-1 [37]. The progeny of this cross, KNA14**a**, was able to mate robustly with the *crg1*Δ mutant of the reference serotype A strain H99α. The deletion of the cryptococcal regulator of G-protein signaling gene, *CRG1*, rendered H99α hypersensitive to pheromone. The **a** progeny of this cross was then able to mate with the wildtype H99α strain, even after crossing out the *crg1*Δ mutation (Figure 1C). Backcrosses with H99α eventually yielded the congenic pair KN99**a**/α. Similar methods could be useful to generate congenic pairs in strains that mate poorly. The availability of the genetically diverse and highly virulent congenic pairs KN99**a**/α (serotype A), XL280**a**/α (serotype D), and AIR265**a**/α (serotype B) allowed further comparison of overall virulence between the **a** and α strains [29,33,37]. These studies found no difference in virulence between **a** and cognate α strains.

The **a** and α coinfection has been used to directly compare the virulence of congenic strains in murine models. In the study performed by Nielsen et al., the mixture of KN99**a** and KN99α cells was used to infect mice intranasally [38]. To easily distinguish **a** from α cells, the strains carried drug markers inserted into the mating type locus at intergenic regions. They concluded that a higher proportion of KN99**a** cells colonize the spleen in a shorter time frame, while KN99α cells colonize the brain more rapidly. One caveat of this study is that the drug marker used was inserted in the *MAT*α locus at a different region compared to the insertion in the *MAT***a** locus due to the idiomorphic nature of the two mating type alleles. To avoid potential complications of using mating-type locus marked strains, Zhai et al. used unmarked wildtype XL280 congenic strains in their coinfection experiments [33]. Given that dissemination from lungs to the brain varies widely among individual mice in the intranasal infection model, they infected one group of mice with the **a**/α mixture intranasally and another group intravenously. They determined the mating type of cryptococcal cells recovered from infected mice through mating with reference strains. At the termination of the intranasal infection group, a significantly higher proportion of α cells were recovered from the lungs, and a higher proportion of **a** cells were recovered from the brain. In contrast, no significant differences were found in colonization of the spleen or kidney. The data from this experiment indicates that the α cells of XL280 may preferentially colonize the lungs. In the intravenous coinfection model, a slightly higher proportion of XL280α cells were recovered from the brain and kidney than XL280**a** cells, and no clear difference was observed in colonization of the spleen when mice were terminated at day 5. A similar coinfection study performed using the AIR265 congenic strains in both intravenous and intranasal models, however, revealed no relationship between the mating type and virulence or tissue tropism [29].

Taken together, subtle differences in tissue tropism may exist in the XL280**a**/α and KN99**a**/α congenic pairs. Because there is no clear correlation between mating type and virulence, it is most likely that the genetic background of individual strains has a significant influence on overall virulence and tissue tropism. The most clear evidence supporting this is the drastic difference in virulence between XL280**a**/α and JEC20**a**/21α, which are sister strains sharing identical mating type loci (Figure 1A,B) [33].

## 6. Examine the Impact of Mating Type on Morphogenesis

While *C. neoformans* is known to undergo **a**-α bisexual reproduction, which leads to production of recombinant basidiospores [19], single isolates can undergo monokaryotic fruiting (or unisexual reproduction) without the participation of the other mating type and yield spores of only one mating type [39]. Monokaryotic fruiting has primarily been reported in serotype D strains, but it also occurs in other serotypes [40,41,42]. Given the sharply skewed distribution of mating types, it has been proposed that a difference in the ability of **a** and α to produce spores during monokaryotic fruiting might have given rise to the dominance of the α mating type. However, monokaryotic fruiting has been observed in isolates of either the **a** or α mating type [43]. Interestingly, among the congenic pairs, α strains showed enhanced ability to undergo self-fruiting compared to the cognate **a** strains, which supports the hypothesis. For instance, JEC21α has the ability to undergo monokaryotic fruiting while its congenic partner JEC20**a** does not [44]. The α mating type of the self-filamentous congenic pair XL280, as well as α progeny of the different backcross generations, showed more robust self-filamentation than the **a** congenic strain and **a** progeny [33]. XL280**a** and XL280α are otherwise phenotypically similar, including virulence in the mouse model, capsule production, melanin synthesis, and resistance to hydrogen peroxide and sodium chloride [33].

Further evidence supporting the contribution of the mating type locus to self-filamentation comes from a quantitative trait loci (QTL) mapping study in 2006 [45]. In this study, Lin et al. isolated an inbred population of 94 serotype D progeny and genotyped them using restriction fragment length polymorphism markers (RFLP). Analysis of the sequence polymorphisms across the genome and the quantitative differences in self-filamentation of these progeny led to the identification of two significant QTLs that highly influence the hyphal length during monokaryotic fruiting. Not surprisingly, the most significant QTL lies in the mating type locus, revealing a positive association of the α mating type with increased hyphal growth. Another significant QTL identified resides in chromosome 9, supporting that self-filamentation is controlled by both genes inside and outside of the mating type locus. Due to the limited number of markers and progeny used, the genes with allelic difference involved in hyphal elongation were not defined in this study. Now with advanced and cheaper genome sequencing technology, higher resolution mapping with a larger population size could help identify the quantitative trait genes located outside of the mating type locus. A bulk segregant analysis strategy that sequences a pool of the filamentous progeny to compare with a pool of the non-filamentous progeny could also be applied to determine the alleles that control self-filamentation.

Thus far, the mechanistic difference in monokaryotic fruiting between the **a** and α mating type has yet to be determined, neither is the influence of the interaction of the mating type locus with other genetic loci. One potential regulator of self-filamentation encoded in the mating type locus is the transcription factor Ste12. In the congenic pair JEC20**a**/21α, the *ste12***a**Δ or the *ste12*αΔ mutant can undergo bisexual mating but not monokaryotic fruiting, and overexpression of either *STE12***a** or *STE12*α induces self-filamentation [46,47]. In comparison, deletion of the *STE12*α gene in hyper filamentous strain XL280α reduces self-filamentation, but does not abolish it [48]. Because XL280α and JEC21α both share the same mating type locus, factors outside of the mating type locus must be influencing the ability to self-filament, echoing the findings from the QTL study.

## 7. Determine the Genetic Factors Contributing to Uniparental Mitochondrial Inheritance

Unlike Mendelian segregation of the nuclear genome where meiotic progeny inherits 50% from each parent, mitochondrial DNA (mtDNA) inheritance during sexual reproduction in *C. neoformans* is uniparental, with most progeny receiving mtDNA from only the **a** parent [49]. Uniparental mitochondrial inheritance was first revealed in inter-varietal crosses between the serotype D JEC20**a** and serotype A α strains because of the ease of distinguishing mitochondrial DNA of different serotypes based on sequence polymorphisms. The congenic pairs JEC20**a**/21α, KN3501**a**/α, KN433**a**/α, and XL280**a**/α all should have identical mtDNA inherited from NIH433**a** (Figure 1). Zhun and Xu later made congenic JEC20**a** and JEC21α strains with different mtDNA, by outcrossing them to strains with different mtDNA and isolating blastospores [50]. The progeny of these strains also showed uniparental inheritance of mtDNA from the **a** parent strain, indicating that uniparental mitochondrial inheritance is not an artifact of inter-varietal mating. The investigations into the molecular control of mitochondrial inheritance have revealed the importance of cooperation between genes located outside of the mating type locus and those inside: The transcription factor Mat2 and regulator Crg1 encoded by genes outside of the mating type locus control multiple genes residing within the mating type locus. The products of these mating type genes are involved in the pheromone-response pathway that drives the formation of the zygote, and the formation of the Sxi1α-Sxi2**a** homeodomain complex that dictates dikaryotic hyphal growth during bisexual mating [51,52,53]. The cooperation between Mat2 and the Sxi1α-Sxi2**a** complex enables strict inheritance of the **a** mitochondria during bisexual reproduction [54]. Uniparental mitochondrial inheritance is demonstrated in crosses of *C. neoformans* isolates and of the *Cryptococcus gattii* VGII congenic pair [29]. However, increased leakage of α mtDNA inheritance was reported in crosses between natural *Cryptococcus gattii* isolates, many of the VGIII lineage [55]. It would be useful to compare mtDNA inheritance patterns among congenic strains constructed in other genetic backgrounds. So far, the evidence suggests that mitochondrial DNA inheritance is controlled by multiple genetic loci, and that differences in their interaction with the mating type locus could be an important source of variation. Detailed discussion on this topic can be found in a separate chapter by Matha and Lin in this Special Issue [56].

## 8. Limitations and Improvement of Congenic Pair Construction

By design, congenic pairs cannot be 100% genetically identical even though they show the same genotype and karyotype based on limited RFLP markers and CHEF electrophoresis. After 10 backcrosses, the progeny in theory are 99.95% identical to the parental backcrossing strain excluding the mating type locus. With a genome size of about 20 Mb, that means a possibility of total regions of the size of 10,000 bp with polymorphisms between the congenic pairs, which could cause phenotypic variations. The observation that progeny of JEC20**a** and JEC21α differed in growth at high temperature, melanization, and capsule production with no clear connection to mating type [57] likely reflects the existence of genetic differences present in other loci. Consistently, comparative analyses of the whole genome sequences of strains JEC20**a** and JEC21α revealed two SNP dense regions other than the mating type locus [57]. One way to prevent misinterpretation of phenotypic differences between the congenic pairs is to include multiple progeny of the congenic pair for phenotypic analyses. Sequencing the genomes of multiple isolates from the 10th backcross could also help pick progeny that are most genetically similar to the backcrossing parent or interpret the observed phenotypic variations. Genome sequencing should become a standard practice to ensure genetic similarity of the congenic pair.

The technical challenge of microdissection of the meiotic basidiospores could cause some issues during congenic pair construction in *Cryptococcus*, particularly in strains where mating is poor. Improving the mating efficiency of *Cryptococcus* through genetic mutations of the mating pathway in the backcrosses could be implemented as in the construction of the congenic pair KN99**a**/α [37]. As long as the mutation is not present in both strains used in a cross, the mutation can be crossed out and will not be present in the final congenic pair. Further study of filamentation and sporulation pathways, therefore, may provide useful tools for this practical purpose.

Another complicating factor in constructing congenic pairs in *Cryptococcus* is the heterogenous nature of mating. Only a small subset of the population is engaged in sexual reproduction and the vast majority of cells amplify mitotically. The meiotic basidiospores are often near mitotically generated yeast cells. Non-recombinant small yeast cells could be mistaken for meiotic progeny either by microdissection or differential centrifugation. If a strain derived from mitotic division from the parents were selected as a progeny and used for the next backcrossing, it would effectively reduce the real number of generations of backcrossing and lead to a higher than expected level of genetic difference among the final congenic strains. Advancement in accurately separating meiotic spores from yeasts would be useful for the construction of congenic pairs, as well as for other applications using any laboratory crosses.

## 9. Concluding Remarks

Congenic pairs are vital tools in genetics research, and they boost the use of *Cryptococcus* as a model organism for basic eukaryotic biology and microbial pathogenesis studies. The current congenic pairs facilitate genetic linkage analyses and the creation of mutants. Their application to *Cryptococcus* research has allowed for the elaboration of the components of the mating type locus and its roles in different aspects of pathogenesis. Mechanisms behind how the mating type locus influences virulence, neurotropism, filamentation, and uniparental inheritance of mtDNA remain to be delineated, and congenic strains may play a crucial role in their elucidation in the future. Construction of congenic pairs in molecular types besides VNI, VNIV, and VGII will help embrace the genetic diversity represented in this species complex and further the investigation of the fundamental biology and pathogenesis in *Cryptococcus*.

## Figures and Tables

**Table 1:** Current *Cryptococcus* congenic pairs.

Pair Name	JEC20/21		KN99		KN3501		KN433		XL280		AIR265	
Recipient Parent	B3502		H99		B3501		NIH433		XL280		R265	
Serotype	D		A		D		D		D		B	
Mating Type	**a**	α	**a**	α	**a**	α	**a**	α	**a**	α	**a**	α
Molecular Type	VNIV	VNIV	VNI	VNI	VNIV	VNIV	VNIV	VNIV	VNIV	VNIV	VGII	VGII
Self-filamentation	-	+	˗	˗	˗	˗	˗	˗	++	+++	-	-
Bisexual Mating	+++	+++	++	++	+++	+++	+++	+++	+++	+++	+	+
Virulence (i.n.)	˧	˧	+++	+++	n/a	n/a	n/a	n/a	++	++	+++	+++
Virulence (i.v.)	+	+˧	+++	+++	+	+	+	+˧	+++	+++	+++	+++
Reference	[19]	[19]	[29]	[29]	[27]	[27]	[27]	[27]	[30]	[30]	[23]	[23]

+: relatively robust ability, ˧: modest ability, -: no ability.
